# Rapid Detection of Adulteration in Dendrobium Huoshanense with Dendrobium Henanense by ATR-FTIR Combined with Multivariate Methods

**DOI:** 10.22037/ijpr.2020.114316.14796

**Published:** 2021

**Authors:** Jing-Wen Hao, Xiao-Quan Liu, Nai-Dong Chen, An-ling Zhu

**Affiliations:** a *College of Biotechnology and Pharmaceutical Engineering, West Anhui University, Lu’an City 237012,China. *; b *Anhui Engineering Laboratory for Conservation and Sustainable Utilization of Traditional Chinese Medicine Resource, Lu’an City 237012, China. *; c *College of Pharmacy, Anhui University of Chinese Medicine, No.1 Qianjian Road, Hefei City, 230012,Anhui Province, P. R. China.*; 1 * J. W. H and X. Q. L contributed equally to this work.*

**Keywords:** Dendrobium huoshanense (DHS), Dendrobium henanense (DHN), Atenuated Total Reﬂectance Fourier transform infrared spectroscopy (ATR-FTIR), Quantitative analysis, PLS

## Abstract

*Dendrobium huoshanense *(DHS) has long been used to make tea drink, soup, and porridge to protect eye and liver in many Southeast Asian countries for centuries. As a rare and endangered functional food, adulteration in DHS with visually similar but cheaper and more accessible plants such as *Dendrobium henanense *(DHN) because of their similarities in morphology has become prevalent in the market. In this study, the Attenuated Total Reﬂectance Fourier transform Infrared Spectroscopy (ATR-FTIR) combined with chemometric methods was established to detect fraudulent addition in DHS with DHN. The partial least squares (PLS) models based on the ATR-FTIR files of DHS mixed with different proportions of DHN were built under cross validation and tested with different independent data sets. To reduce the variables’ lack of information and increase the accuracy of the model, diﬀerent wavelength selection methods including Moving Window Partial Least Squares (MW-PLS), Monte Carlo-uninformative variable elimination (MC-UVE), and interval random frog (iRF) were compared.The results showed that iRF performed the most perfectly with the number of latent variables (nLVs = 7), the lowest Root Mean Square Error of Cross-Validation (RMSECV = 7.37), and the maximum determination coefficients (R^2 ^= 0.9721). The excellent performance of the model was proved by the low RMSEP value of 6.44% and the high R^2^ value of 0.9556. The developed method could rapidly quantify the adulteration DHN in DHS, and our study might provide an efficient and great potential technique tool for the rapid, green, low-cost, and nondestructive identification and quantification for DHS adulterated with DHN.

## Introduction

**Figure 1:**
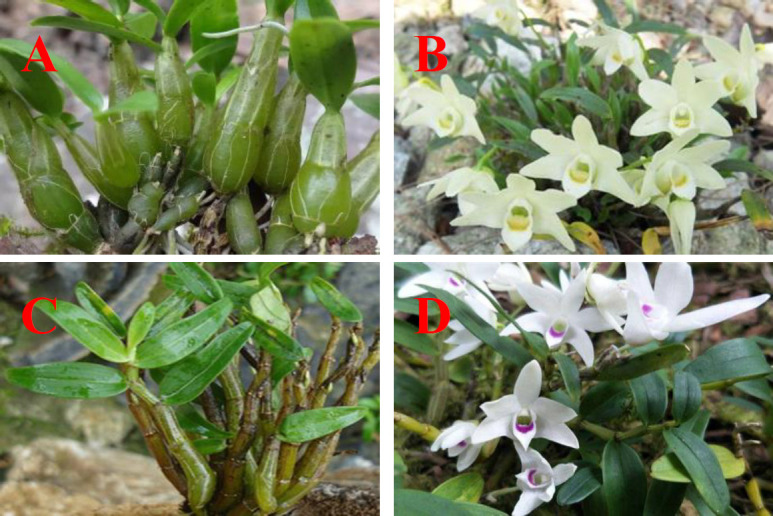
*D.huoshanese* and *D.henansese.*A: *D.huoshanese*; B: the flower of *D.huoshanse*; C: *D.henanese*; D: the flower of *D.henanese*

*Dendrobium huoshanense *C. Z. Tang and S. J. Cheng (DHS) (Figure 1), a rare and endangered edible-medicinal plant of the Orchidaceae family, have been used as a tonic or a functional food to improve body health and ophthalmic disorders, to prevent cataract, throat inﬂammation and chronic superﬁcial gastritis for centuries in China (1, 2). DHS has also long been used as a food material to make tea drinks, soup, and porridge for the protection of eye and liver in many Southeast Asian countries (3-6). The strong demand and the low yield made the selling price of DHS even reach RMB 100-150 thousand yuan per kg in the markets of Chinese medical materials in China in 2019. 

Consequently, adulteration with visually similar but less expensive and more accessible plants has become prevalent in the market. Market snapshots found that DHS and its products were commonly admixed with *Dendrobium henanense* (DHN) (Figure 1) because of their similarities in morphology while much cheaper (about RMB 5-6 thousand yuan per kg) and more abundant resource of DHN (7-9). Discrimination of DHS and DHN by visual inspection is quite difficult without the aids of instruments, even by the professionals. Developing a rapid, green, low-cost, and nondestructive method the rare DHS adulteration with DHN is urgent. 

Recently, studies about the determination of adulterations in foods using spectroscopic methods have been extensively carried out (10-12). Fourier transform infrared spectroscopy (FTIR) is becoming a desirable candidate because of its high specificity, convenience, short processing time, low cost, and nondestructive properties (13). In a previous study, we have successfully applied the FTIR, 2D COS IR for the discrimination of different ages and origins of *Dendrobium* plants and achieved to establish ATR-IR and ATR-NIR quantification models to determine the polysaccharide and polyphene in DHS (2, 14). However, the application of ATR-FTIR spectroscopy combined with chemometrics to detect the DHS adulteration has not been previously reported.

This study aimed to investigate the possibility of using ATR-FTIR spectroscopy combined with chemometric data analysis as a rapid and easy tool for the determination of DHS mixed with different contents of its common adulterant DHN. 

## Experimental

*Materials*

A total of 210 samples, including 10 original DHS samples, 10 original DHN, and 190 adulterated DHS samples with the known adulteration levels, were prepared in laboratory conditions as explained in the following subsection. 

*Preparation of the synthetically adulterated DHS samples*

All samples were cut into small pieces and freezing-dried at -50 C and then pulverized and passed through a 60-mesh sieve (particle size-0.2 mm) under laboratory conditions. The 190 adulterated samples were prepared by mixing DHN with pure intermediate ultraﬁne powder of DHS to ﬁnal concentrations (w/w) of 5%, 10%, 15%, 20%, 25%, 30%, 35%, 40%, 45%, 50%, 55%, 60%, 65%, 70%, 75%, 80%, 85%, 90%, and 95%, with ten repetitions per level. Approximately 500 mg of each sample was prepared and kept at -20 C until the ATR-FTIR analysis.

The ATR-FTIR spectrum of all the samples were used to establish PLS models for the quantitative analysis of DHN in adulterated DHS sample.

*Collection of FTIR spectra of DHS samples*

The spectra of the samples were recorded in the MIR range of 400-4000 cm^−1^ with a resolution of 4 cm^−1 ^using a Nicolet iS50 FTIR spectrometer equipped with a diamond single reflection ATR accessory equipped by a diamond as a device manager (Thermo Scientiﬁc, Waltham, MA, USA). Each spectrum was collected for 32 scans in the absorbance mode. The mean spectrum of triplicate measurements was used as the FTIR spectrum of each sample.

The software OMNIC version 8 and Thermofisher Quantity Analyst 8 (Thermo Fisher Scientific) were used for spectral acquisition and further analysis, respectively. 

*FTIR spectral pre-processing*

The spectral pre-processing is extremely important to reduce the interference and undesirable information and improve the contribution of chemical gradients. In this paper, different data pre-processing techniques including MSC, SNV, the first derivative and the second derivative by the Savitzky-Golay algorithm (15 data points), and the combinations of MSC with the first derivative, the SNV with the first derivative, MSC with the second derivative, and the SNV with the second derivative, were investigated to get reliable, accurate and stable models, because the background information and noise among the sample information are involved in the raw FTIR spectra . Then the results of eight different signal pretreatment methods were evaluated and compared.

*Samples division method*

The TQ-analyst 8 selection of training and test samples separately in classes. Thus, the samples were split into 105 (10 DHS samples, 95 DHS samples adulterated with DHN) for the training subset and 105 (10 DHS samples, 95 DHS samples adulterated with DHN) for the test subset. 

*Wavenumber selection method*

Three different wavelength selection methods involving moving window partial least squares (MW-PLS), Monte carlo-uninformative variable elimination (MC-UVE), and interval random frog (iRF) were compared to obtain the effective wavelengths for the establishing of PLS models. 

*MW-PLS*

As a wavenumber interval selection method, MW-PLS could make the PLS calibration model stable and avoid interference from the factors of irrelevant constituents (13). In this paper, the favourable spectral interval of the samples was chosen by the lowest Root Mean Square Error of Cross Validation (RMSECV), which could be calculated as following Equation 1. 

$\mathrm{RMSECV}=\sqrt[{2}]{\frac{1}{n}\sum_{i=1}^{n}{\left(y_{\mathrm{real}}-y_{\mathrm{pre}}\right)}^{2}}$

 Equation 1.

where y_real_ is the actual value of the investigated sample, y_pre_ is the predicted value of the investigated sample. Generally, the lower RMSECV represents more information of the selected wavenumbers.

*MC-UVE*

Applying the MC-UVE, calibration samples are chosen arbitrarily to establish a chain of PLS models during each Monte Carlo (MC) sampling at first. Then the stability of each wave number is evaluated according to the PLS regression coefficient matrix during model calibration. To each MC procedure, the PLS regression coefficient is expressed as b = (b_1_, b_2_,…., b_j_…., b_p_). After M simulations, a PLS regression coefficient matrix B = (B_1_; B_2_;….B_M_) can be acquired. Since each b_j_ represents the contribution of the j-th wave number to the PLS model, the stability of each wavenumber can be calculated by Equation 2. 

$S_{j}=\frac{\mathrm{mean}(b_{j})}{{\mathrm{std}}_{{(b}_{j})}}$

 Equation 2.

where mean (b_j_) and std (b_j_) are the mean and standard deviation values of the regression coefficient at j-th wave number in the PLS model.

According to the stability of all the wavenumbers, the wavenumbers, the stability value of which below the cut-off values, will be deleted by the last classification performance. 

*iRF*

iRF is a wavelength interval selection method that considers the continuity of spectra (15). Applying iRF, spectra are first fractionated into sub-intervals of the whole spectra by a moving window of a fixed width, and thus it can gain all of the possible continuous spectral intervals. The adopting possibility of each variable after N iterations is computed. The frequency of the j-th variable, j = ( 1, 2,….,p) chosen in these N variable subclasses, is expressed as *N*_j_*.* The adopting possibility of each variable can be accounted by Equation 3.

$\mathrm{Probability}j=\frac{N_{j}}{N}$

 Equation 3.

In this paper, the best intervals with the lowest RMSECV are chosen. The width of the interval was set to 20 due to 7467 full spectral points, and this resulted in 7467 intervals, and each interval obtained 20 variables. 

*Quantification models with PLS*

Partial least square regression (PLS) is one of the most common regression algorithms in chemometrics in general, particularly for spectroscopy.

The PLS models were evaluated by leave-one-out cross-validation by mapping the number of factors against the RMSECV. The performance of the PLS models was commonly evaluated by the determined coefficients (R^2^) and RMSECV. In addition, the root-mean- square error (RMSE), which respectively expressed as root-mean-square error of calibration (RMSEC) for calibration set and as root-mean-square error of prediction (RMSEP) for test set in this experiment, was used to appraise the established PLS model. A superior model should show high R^2^ values while low RMSECV, RMSEC, and RMSEP (2). 

## Results and Discussion

*Spectral comparison analysis*

**Figure 2:**
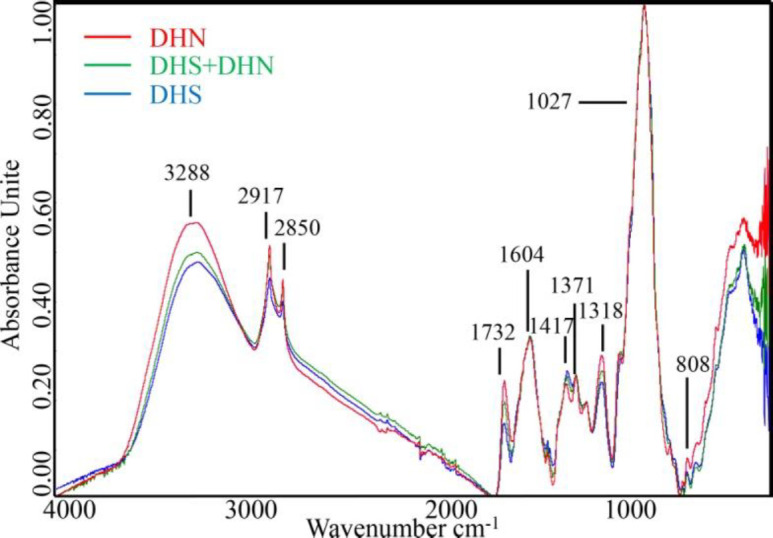
Raw ATR-FTIR spectra from the experiments of pure DHS, pure DHN and representative adulterates samples

**Figure 3:**
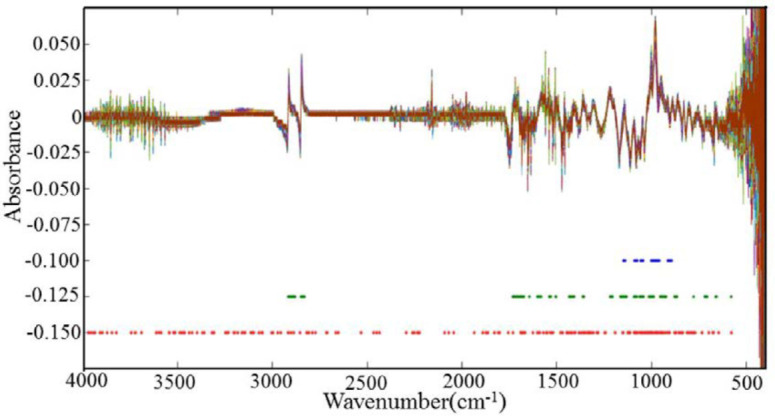
The distribution of the selected variables obtained using different wavelength selection methods. Note: The blue dots represent the wavelength values obtained by moving window PLS (MW-PLS) method, the green dots represent the wavelength values obtained by Monte Carlo Uninformative Variable Elimination (MC-UVE) method, the red dots represent the wavelength values obtained by interval Random Frog (iRF) method

Figures 2 and 3 exhibited the raw FTIR spectra of DHS and its adulterated samples. As it can be seen, the spectra of all samples presented a broad and intense band in the region from 3600 to 3000 cm^-1^ related to the hydroxyl group bands absorption of water (9, 16). Further analysis of the FTIR files revealed that there were two sharp peaks at approximately 2917 cm^-1^and 2850 cm^-1, ^both related to the alkyl group absorption (16), 4 relatively strong characteristic absorptions of C-O stretching vibrations about peaks at approximately 1732 cm^-1^, 1604 cm^-1^, 1371 cm^-1^, 1318 cm^-1 ^(17, 18). The peaks at approximately 1417 cm^-1^ showed a combination of bands of low intensity that could be related to CH_2 _(19). The peaks at approximately 1027 cm^-1^suggests the C-O-C link bond, and the peak at approximately 808 cm^-1 ^showed bending vibrations of C-H bond (20). However, the spectra of authentic, pure DHS, pure DHN, and the DHS adulterate different degrees of DHN showed similar trends by vision (Figure 2), and it is challenging to directly distinguish the adulterate samples only by the FTIR spectrum. Accordingly, advisable spectral pretreating is essential to outstand the differences caused by adulterants. 

*Spectral pre-treatment*

PLS spectrum model was established with different data pretreating methods. In this paper, 21-fold cross-validation was applied to select the number of latent variables and the optimal spectral data pretreating methods adopting the whole sample set (S1-S210). The spectral pretreating was optimized based on the lowest RMSECV and the highest *R*^2^. According to Table 1, comparing the PLS model based on the original FTIR spectrum, data pre-processing could remarkably increase the accuracy of the PLS model. Among the models based on the 8 spectra pre-processing methods, the best one was built with data pretreated by SNV+first derivative due to its lowest RMSECV = 9.58 and highest R^2 ^= 0.9487 for the real DHN contents in DHS samples.

 When there are overlapping peaks in the raw FTIR spectra, SNV+first derivative for the data pre-processing is generally favorable to improve the resolution. As shown in Figure 3, most of the absorbance values were nearly zero, and the overlapping peaks were separated after spectral pretreatments. 

The spectra of DHS and adulterated DHS pre-processed by taking SNV+first derivative procedures are shown in Figure 3. No feature peak is directly related to the adulterants in the SNV+first derivative spectra, and it is still difficult to discriminate the spectrum between DHS and DHS adulterated different degrees of DHN only by the direct observation. Therefore, chemometric methods are still needed for data mining, qualitative and quantitative analysis. 

*Wavelength selection models*

To get selection behavior of different wave number selection models, MW-PLS, MC-UVE and iRF are employed to select the representative wave numbers of the spectra of DHS and DHS adulterated different degrees of DHN. Comparing the full spectrum model, the model based on the best-selected wavelengths should obtain the lowest RMSECV, the smallest number of wavelengths. The calibration and validation results of each wavelength selection model were shown in Table 2.

As it was shown in Table 2, the RMSECV, R^2,^ and nLVs of the prediction of the full spectrum model were 9.58, 0.9487, and 10, respectively. All the other wavelength selection models carried out better than the full spectrum PLS model according to the RMSECV, R^2,^ and nLVs. In addition, the number of selected wavelengths of MW-PLS, MC-UVE, and iRF were 190, 603, and 238, which are also much less than the 7467 wavelengths of full spectrum. Therefore, the PLS model can perform well if the uninformative variables and irrelevant information were eliminated. 

MW-PLS and iRF is a wavelength interval selection method, while MC-UVE is the discrete wavelength selection methods. All of them are based on the PLS regression coeﬃcients. In our study, to determine the DHN contents in powder samples, the results showed that iRF obtains the best performance with the lowest RMSECV, the highest R^2, ^and the least nLVs among the three-wavelength selection methods. Thus iRF was chosen as the wavelength selection method in the later establishment of the PLS models. 

As plant materials, DHN and DHS contain lots of compounds with different functional groups with complex linkages. In order to understand and interpret the selected wavelengths in all of the wavelength selection methods, they are displayed in Figure 3. The wavelengths selected by MW-PLS are very concentrated (800-1200 cm^-1^), resulting in MW-PLS performing a little better than the full spectrum model. MW-PLS, MC-UVE, and iRF have some commonly selected regions. As iRF performs the best in this work, the interpretation of the selected wavelengths focuses on iRF. We can see that the wavelengths selected by iRF are mostly concentrated in the region 600-1800 cm^-1^. In addition, other regions were also used, which have provided more useful information for iRF. 

From the above points, it can be proven that wavelength selection is necessary in multivariate calibration for the FTIR analytical system.

*Quantiﬁcation model*

A quantiﬁcation model based on the PLS was constructed for the determination of the DHN contents in the adulterated samples. For this determination, the samples were divided into calibration and validation sets as described. To ensure the quality of the variable selection algorithms, the development of a total DHN content prediction model was ﬁrst accomplished by spectrum PLS. 

**Figure 4:**
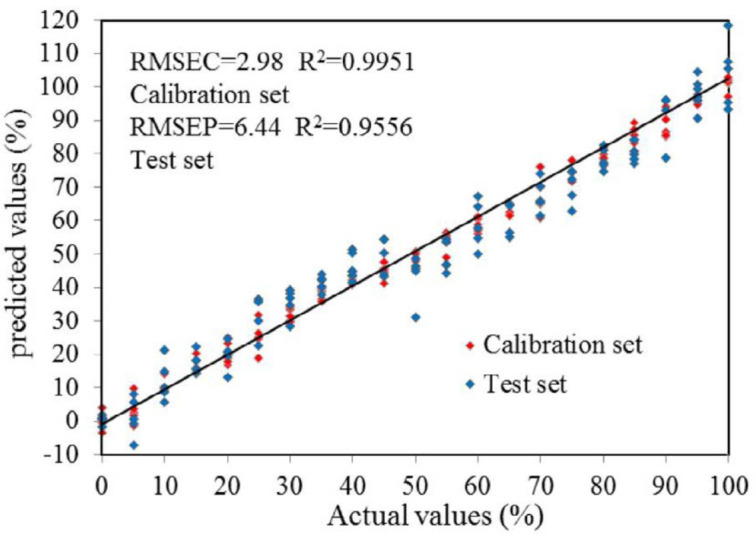
The results was comparison of the real adulterate contents in DHS samples with DHN and those predicted by the established infrared calibration models

Adopting the best spectra pretreatments and the wavelength selection method, the PLS models were developed. Figure 4 showed the correlation between the real contents of DHN and those predicted by the established FTIR models. It is thus evident that the results assayed by the FTIR model were entirely consistent with those calculated. Linear tendencies were perspicuous, and each plot obtained a gradient of unity and a zero intercept. The R^2^ and the RMSEC for the FTIR model were 0.9951 and 2.98, the test validation to verify the robustness of the developed quantification FTIR models to predict an independent test set of sample. The RMSEP of 6.44 and the *R*^2^ of 0.9556 were achieved for the contents of DHN by FTIR models; this displayed that the calibration model was quite excellent and accurate. 

**Table 1:** Effects of pretreatment methods on performance (R^2^, RMSECV) of PLS calibration model

**Pretreatment methods **	**PLS results for DHN**	
	**R** ^2^	**RMSECV**
Original	0.9344	11.0
MSC	0.9441	10.0
SNV	0.9451	9.94
first derivative	0.9350	10.8
second derivative	0.4823	33.8
MSC+first derivative	0.9438	9.75
MSC+ second derivative	0.3079	32.4
SNV+first derivative	0.9487	9.58
SNV+ second derivative	0.3259	32.4

**Table 2:** Results of DHN contents using PLS models applied different wavelength selection models

	**Full specerum**	**MW-PLS**	**MC-UVE**	**iRF**
N.W^a^	7467	190	603	238
nLVs	10	1	3	7
RMSECV	9.58	8.40	8.23	7.37
R^2^	0.9487	0.9608	0.9630	0.9721

^a^N. W is the number of wavelengths.

## Conclusion

In this work, we succeeded proposing an analytical methodology to discriminate authentic DHS, and its adulterates, to detect the adulterated DHN contents in adulterates using ATR-FTIR spectroscopy combined with chemometric methods. from the results of this study indicated that appropriate spectra pre-processing selection modes and proper wavelength selection methods could significantly increase the predictive accuracy of FTIR PLS models. FTIR spectroscopy with the PLS algorithm could be applied to determine the content of total DHN in ultraﬁne granular powder of DHS adulterates. FTIR spectroscopyand chemometric methods can offer a simple, fast and reliable method for the adulterate of the DHS, and might provide reference for the adulterate of other TCMs.
